# Editorial

**Published:** 1864-03

**Authors:** 


### Editorial.

### NITROUS OXIDE AS AN ANÆSTHETIC.

The query comes to us very often, "What about Nitrous Oxide?" "Is it safe?" "Is it practicable?" "Is it efficient?"

In regard to its use, some are using it extensively, and think very highly of it, and think it not dangerous if used properly, some have used it in thousands of cases without perceiving any unfavorable syptoms, while some others see or assume to see difficulties and dangers at every move. Comparatively few in the profession are using it or ever will use it, at least in the present mode of preparation and administration. There are many in the profession who, with their present knowledge of chemistry and its manipulations ought not to attempt its use, and further, none should attempt to use any anæsthetic who does not well understand physiological and pathological conditions, and manifestations. No one should assume to use chloroform or ether, who can not make a definite and accurate test of their purity. With many of us their is a total want in regard to chemistry and its manipulations. Others again have a limited knowledge, but not enough to warrant them in preparing and administering anæsthetics.

In regard to the safety of Nitrous Oxide, we think there is not much to fear, if properly employed; of course life may be destroyed by almost any thereputic agent—even good beefsteak has destroyed life—and certainly by any agent whose influence and effects are as extensive and rapid as Nitrous Oxide, Chloroform, or Ether, life may easily be destroyed; but the same is true of Opium, Mercury, Arsenic and Strychnine, yet they are used constantly, by those who understand them. The wholesale destruction of life that seemed about to take place by the influence of Nitrous Oxide is pretty effectually dissipated by a communication which we publish on another page of this number.

We should not hesitate to use this agent as an anæsthetic on the score of the want of safety, but in the present mode of administration, we regard it impracticable for general use. It may be used with entire success when the whole attention is given to it, but its use is hardly admissible in a full general practice. The present mode of administering is cumbersome, and forbidding in appearance, besides it is objectionable for all to inhale from the same sack or gas-holder. We hope some better mode of administering will be introduced.

The Nitrous Oxide has some advantages over Chloroform or Ether. In its use the operator is entirely free from its influence—would that this much could be said of Chloroform or Ether.

In its mode of action upon the nervous system, it is, in our opinion far less objectionable than Chloroform or Ether. The patient on recovering from it, feels better than before taking it; there is a delightful vivacity and buoyancy experienced, and in no case have we known of nausea or headache accompanying it, one or both of which, very frequently occur, especially from the use of Ether.

We have used Nitrous Oxide, but little, and so far are favorably impressed with its action. We shall have more to say upon the subject ere long. T.

### GOING TO PARIS.

Our friend and professional brother, Dr. H. J. McKellops, of St. Louis, is about going to Paris, with a view of entering upon the practice of his profession. And while we regret to loose him—our profession can not well afford to loose such men—we are proud to have such a representative in Europe. We know Dr. McKellops will make his mark there, and a mark for our profession too, that every one will be gratified to recognise, and acknowledge. He will not be forgotten by those he has left behind, neither will he forget them, but will place them in a proper light before those with whom he may come in contact. He is one of the finest operators in the profession; success will attend him wherever he goes, for this he has our earnest prayer. The readers of the Register will hear from him. T

### AUTOMATIC PLUGGER.

We have seen, and used a little, an instrument which we denominate as above. It is designed to take the place of an assistant in the use of the mallet.

It is quite an improvement upon anything heretofore suggested or presented. It is far more compact, less complicated and more efficient, than anything of the kind we have seen. We shall, perhaps, give a cut of it in the next number of the Register. It is the invention of Dr. E. Collins. of Richmond, Ind. T.

### DENTAL DEPOT.

Dr. J. S. Walter & Co., recently of Rochester, N. Y., have purchased the Toland Dental Depot, of this city, and have replenished it with new stock of all kinds to overflowing; they have rearranged and set in order the whole establishment, and we feel no hesitation in saying, that they will keep a dental stock, in every way worthy of the patronage of the profession of the West. We have been personally acquainted with Dr. Walter for many years, and know him to be eminently qualified for the position which he has assumed. He was a dentist in extensive practice in western New York for a long time, and knows perfectly the wants of the profession, and well understands how to meet them. His knowledge is such that we shall be glad to consult him frequently in regard to dental materials. T.

### THE OHIO DENTAL COLLEGE ASSOCIATION.

This Association met in the lecture room of the College on the 20th of February ult. Much business of importance to the Institution was transacted. Subscriptions of stock were obtained by which the College is entirely released from pecuniary embarrassment; and such arrangements are made as will give it the most complete outfit possible in every department, and we hope that with the new impetus given, and the interest manifested by the members of the Association, the Institution will fully meet the highest expectation of its friends and the profession.

